# Interpersonal Functioning in Borderline Personality Disorder Traits: A Social Media Perspective

**DOI:** 10.1038/s41598-020-58001-x

**Published:** 2020-01-23

**Authors:** Jinnie Ooi, John Michael, Sakari Lemola, Stephen Butterfill, Cynthia S. Q. Siew, Lukasz Walasek

**Affiliations:** 1 Department of Philosophy, University of Warwick, Warwick, UK; 2 Department of Cognitive Science, Central European University Budapest, Budapest, Hungary; 3 Department of Psychology, University of Warwick, Warwick, UK; 4 Department of Psychology, National University of Singapore, Singapore, Singapore

**Keywords:** Social behaviour, Human behaviour

## Abstract

This is the first study to demonstrate interpersonal difficulties associated with borderline personality disorder (BPD) features in the domain of social media. Using crowdsourcing, we presented participants with a battery of questions about their recent social media use, and then assessed their BPD features using the short form of the Five-Factor Borderline Inventory. The results revealed that individuals with higher BPD trait scores reported posting more often on social media, as well as a higher incidence of experiencing regret after posting on social media, and of deleting or editing their posts. They also report a higher degree of importance of social media in their social behavior and daily routines. These results highlight the pervasiveness of interpersonal difficulties associated with BPD features even in the non-clinical population, and demonstrate that these difficulties are also observable in social media behavior. Our findings may provide a starting point for research using data from social media to illuminate the cognitive and emotional processes underpinning the interpersonal difficulties associated with BPD features, and to inform and assess therapeutic interventions.

## Introduction

Borderline personality disorder (BPD) is a severe psychiatric condition associated with significant psychosocial impairments, high rates of comorbidity with other psychiatric conditions^1^, high rates of suicide^2^ and considerable economic costs due to intensive use of treatment and loss of productivity^3^. It is marked by conflicted relationships, difficulty trusting other people, fear of abandonment, and patterns of overinvolvement/withdrawal as well as idealization/devaluation of relationships^4^. In more general terms, *impairment in interpersonal functioning* has been highlighted as a core feature of psychopathology in BPD, alongside *affect dysregulation* and *behavioral dysregulation* (in particular impulsivity)^5^.

In addition to the clinical population – which makes up 2–4% of the general population^6,7^– persistent impairment in interpersonal relationships has also been reported in non-clinical populations exhibiting high levels of BPD features^8,9^. Thus, researchers have recently turned their attention to investigating the behavioral manifestations of BPD features in non-clinical populations. For example, it has been shown that individuals with BPD features exhibit deficits in emotional understanding and management of both their own and others’ emotions^10,11^. And it has also been found in one recent study^12^ that people’s sense of commitment in joint activities and relationships may be influenced by BPD features. Thus, we may expect to observe the difference in the emotional functioning of people with BPD features even without the presence of diagnosis of BPD.

And yet there has been very little research so far investigating the behavioral manifestations of BPD features in the domain of social media. This is surprising insofar as social media constitute an increasingly central arena of social interaction for people in general^13^. Indeed, we may expect this to be all the more true of individuals with high BPD features, given that the difficulties they typically experience with interpersonal relations may lead them to be particularly motivated to engage with social media in order to satisfy a pronounced need for social connection. In one of the few studies looking at BPD features within this domain, elevated risks of problematic Facebook use (addictive behaviors) were found in a group of adolescents and young adults exhibiting a ‘borderline’ profile (defined as showing a combination of high borderline personality features, depressive symptoms, social anxiety, and sensation seeking^14^). It is unsurprising that *maintaining social relationships* was reported as one of the main motives for Facebook use in this study, plausibly to maintain a compensatory level of social engagement in those with limited real-world social networks. However, it remains unclear whether the interpersonal difficulties that individuals with high BPD features experience are also displayed in social media behavior.

The present study addresses this challenge by examining how the interpersonal dysfunction associated with high BPD features in non-clinical samples is expressed in the domain of social media. Specifically, we tested the following predictions:

P1) Given that interpersonal dysfunction is associated with persistent loneliness in BPD^15^, we reasoned that individuals with higher BPD trait scores may feel more of a need for social connection through social media use, and therefore predicted that they would (P1) report posting more often on social media (in the previous two weeks).

P2) Because high levels of BPD features are associated with impulsivity and with interpersonal dysfunction, we hypothesized that individuals with higher BPD trait scores would more often regret actions which they perform on social media (e.g., making posts, tweeting). We therefore predicted that they would report (P2a) a high incidence of experiencing regret after posting on social media, and (P2b) delete/edit their posts more frequently.

P3) Due to this interpersonal dysfunction, social network analysis revealed that individuals diagnosed with BPD were more likely to cut off (stop speaking to) social partners than non-PD clinical controls (31% vs. 9% in the past year respectively)^16^. Thus, we predicted that individuals with higher BPD trait scores would (P3) more often show similar ‘cutting off’ behaviors on social media, namely unfriending or blocking other users.

P4) Given that individuals diagnosed with BPD report having smaller social networks (i.e., fewer social partners) compared to healthy controls^15,17^, we predicted that individuals with higher BPD trait scores would (P4) have smaller virtual social connections (e.g., fewer friends on Facebook, followings and followers on Instagram).

P5) Finally, we predicted that Individuals with higher BPD trait scores would (P5a) spend more time using social media and (P5b) show greater integration of social media in their social behavior and daily routines.

The predictions, sample size, methods, and planned analyses were all pre-registered before data collection and can be accessed at: https://aspredicted.org/cq83y.pdf.

## Results

We report our results following analysis plan specified in our pre-registration. In the case of count data, we selected between a Poisson model and negative binomial model on the basis of the Vuong’s non-nested test^18^. In addition to the standard frequentist analysis, we also report results of the equivalent Bayesian model which we implemented using brms package in R^19^. In each model, we used a normally distributed population-level priors with mean of 0 and standard deviation of 10. Our model run with four chains, each with 2000 iterations (1000 warm-up).

**Figure 1:**
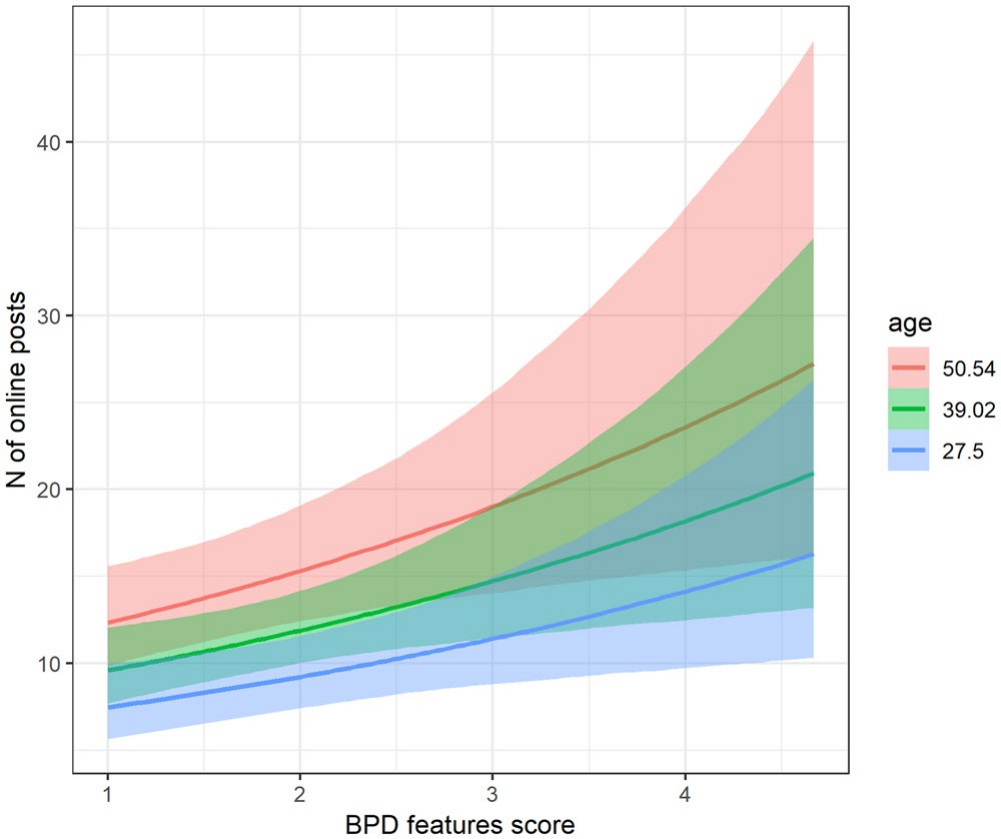
Marginal effects of BPD trait score and age on the self-reported number of online posts.

Our first prediction (P1) was that people with higher BPD trait scores would report posting more often on social media (in the previous two weeks). Results of the negative binomial test support this hypothesis, with a positive and significant coefficient of BPD trait scores, *β* = 0.21, 95% CIs [0.06; 0.37], *p* = 0.012. Notably, we also found a significant effect of age, such that older individuals posted more frequently on social media, *β* = 0.02, 95% CIs [0.01; 0.03], *p* < 0.001. There were no outliers based on the Bonferroni outlier test^20^. Results of the Bayesian model were consistent with these findings. Confidence intervals for BPD trait scores, *β* = 0.21, 95% CIs [0.06; 0.37], and age, *β* = 0.02, 95% CIs [0.01; 0.03], did not include zero. Figure 1 illustrates marginal effects from the Bayesian analysis, with separate lines for different ages.

**Figure 2:**
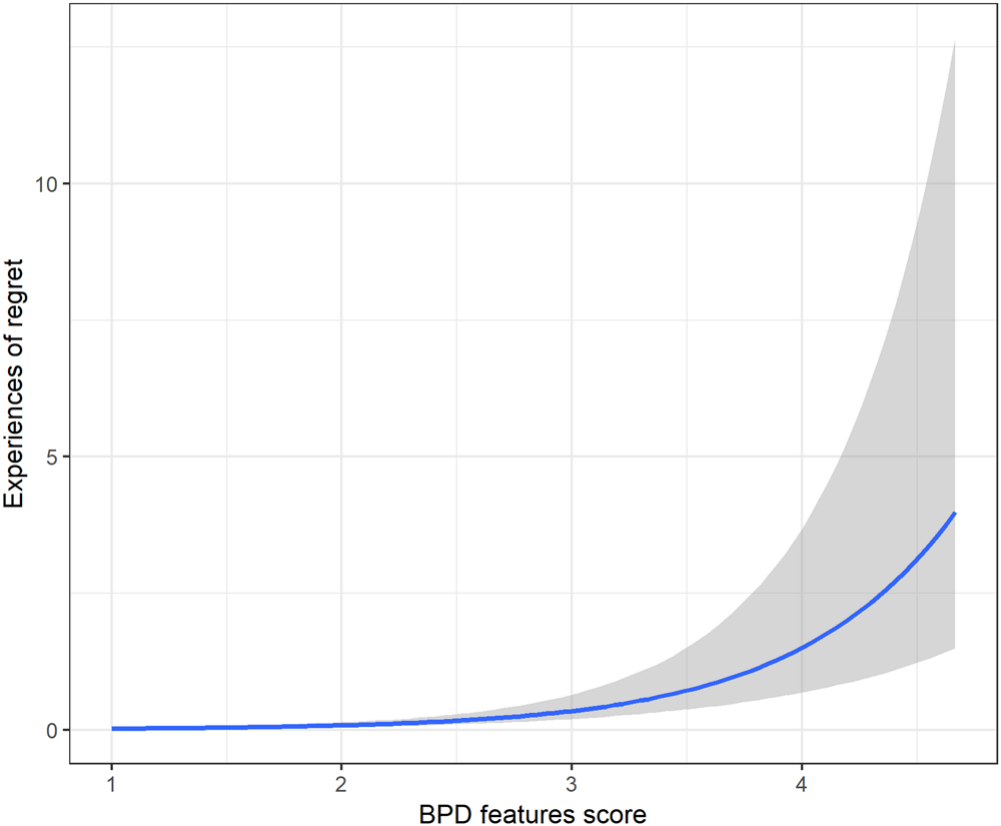
Marginal effects of BPD trait score on the self-reported number of instances of regretting a post on social media.

**Figure 3:**
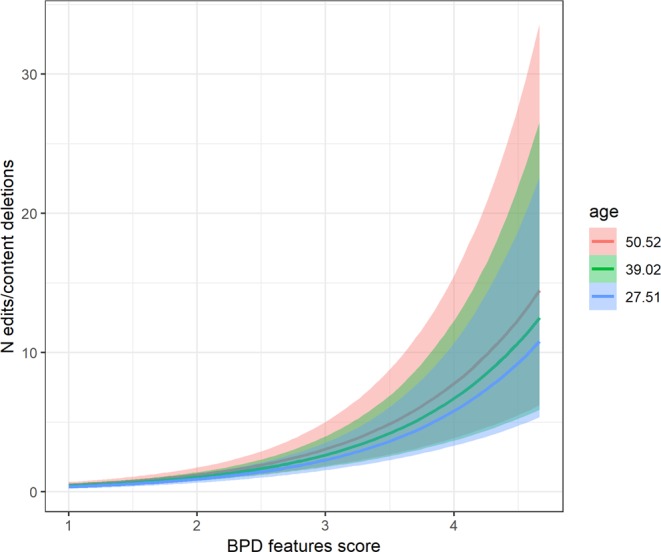
Marginal effects of BPD trait score on the self-reported number of instances of editing or deleting a post on social media.

Our second hypothesis (P2) was that participants with higher BPD trait scores would report experiencing a) more regret after posting on social media more often, and b) delete/edit their posts more frequently. Both hypotheses were supported. For regret, the coefficient of the BPD trait score was positive in the frequentist, *β* = 1.43, 95% CIs [1.07; 1.84], *p* < 0.001, and Bayesian analysis, *β* = 1.47, 95%CIs [1.09; 1.88] (10 outliers removed). For deleting/editing, the coefficients were also positive in frequentist *β* = 0.92, 95% CIs [0.67; 1.17], *p* < 0.001, and Bayesian versions of the test, *β* = 0.93, 95% CIs [0.68; 1.18]. Note that neither the effect of gender nor age were significant in the case of regret, but we found a positive effect of age and a negative effect of gender (males scoring lower with higher BPD trait score) for the frequency of editing and deleting. Marginal effects for P2a and P2b are shown in Figs. 2 and 3, respectively.

**Figure 4:**
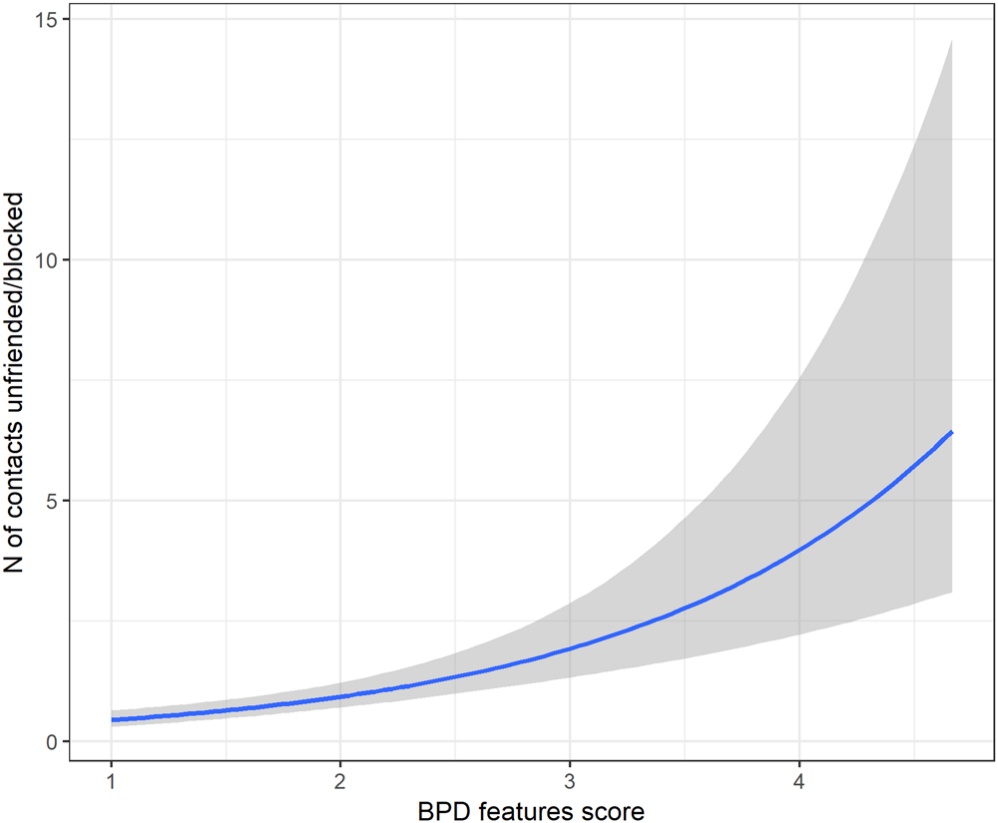
Marginal effects of BPD trait score on the self-reported number of occasions in the past month on which respondents blocked or unfriended other users.

Our third hypothesis (P3) concerned the frequency with which individuals unfriend, mute and block other users on social media. The composite (sum) score of these individual variables was tested using a negative binomial test. Consistent with our prediction, there was a positive relation between BPD trait scores and rejecting behaviors on social media, *β* = 0.72, 95% CIs [0.47; 0.98], *p* < 0.001 (no outliers). Neither the effect of age or gender were significant. The Bayes analysis showed similar results, *β* = 0.73, 95% CIs [0.48; 1.00]. Marginal effects are shown in Fig. 4.

Our fourth hypothesis (P4) was that people with higher BPD trait scores would have smaller social circles on social media platforms. Specifically, we were interested in the number of friends on Facebook, as well as the number of followers and people followed on both Instagram and Twitter. For each of these variables, we report individual tests for those individuals who used these social media platforms. Table 1 below summarizes the findings of the frequentist and Bayesian tests. Overall, we do not find support for our hypothesis, as there appears to be no clear relationship between BPD trait scores and the numbers of social media contacts. However, we found two small and significant effects for the number of people following a given user, and the number of accounts followed by our participants.

**Table 1:** Summary of the findings of the frequentist and Bayesian tests.

Dependent variable	N	Negative binomial regression	Bayesian Negative binomial regression
N Facebook friends	505	*β* = −0.04, 95% CIs [−0.15; 0.07]	*β* = −0.04, 95% CIs [−0.15; 0.08]
N Twitter following	351 (1)	*β* = 0.23, 95% CIs [−0.03; 0.44]	*β* = 0.24, 95% CIs [0.03; 0.45]
N Twitter followed by	351 (4)	*β* = 0.27*, 95% CIs [0.06; 0.48]	*β* = 0.27, 95% CIs [0.06; 0.49]
N Instagram following	281	*β* = 0.25*, 95% CIs [0.04; 0.46]	*β* = 0.25, 95% CIs [0.04; 0.46]
N Instagram followed by	280	*β* = −0.01, 95% CIs [−0.22; 0.22]	*β* = 0.00, 95% CIs [−0.21; 0.23]

Numbers in brackets in the *N* column indicate the number of outliers removed from the sample. Note. *p < 0.05, **p < 0.01, ***p < *0.001*.

We also hypothesized (P5a) that people with higher BPD trait scores would spend more time (here minutes in a day) on social media platforms. We used the log transformed total time and conducted a linear regression with BPD trait score, age and gender as predictors. As per our pre-registration, we removed extreme values (+−1.5 * Interquartile Range), which left us with 576 responses. The results revealed no association between BPD trait scores and time spend on social media, *β* = 0.009, 95% CIs [−0.008; 0.03], *p* = 0.285. The Bayesian result was consistent with this conclusion, *β* = 0.01, 95% CIs [−0.01; 0.03]. Neither effect of gender nor age were significant.

**Figure 5:**
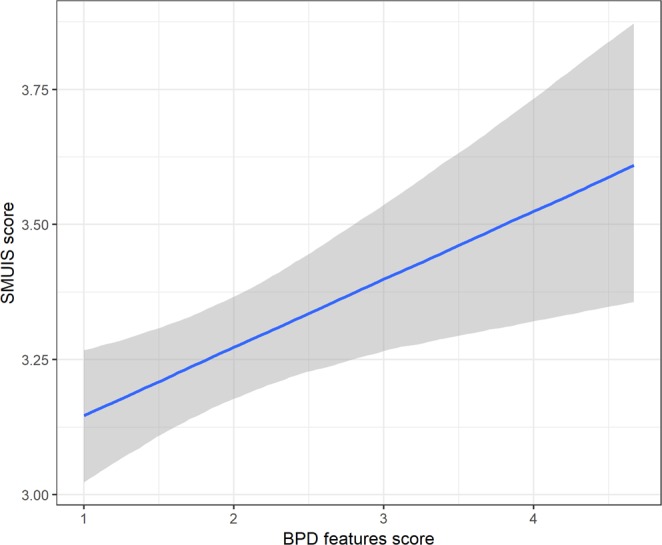
Marginal effects of BPD trait score on self-reported ratings on the Social Integration and Emotional Connection subscale of Social Media Use Integration Scale^17^.

Finally, we expected that for people with higher BPD trait scores, social media would be a more integral part of their lives and daily routines (P5b). A linear regression supported this prediction, showing a positive association between BPD trait scores and scores on the SMUIS scale, *β* = 0.13, 95% CIs [0.04; 0.22], *p* = 0.006 (no outliers detected). The results of the Bayesian produced the same result, *β* = 0.13, 95% CIs [0.04; 0.22]. While we did not find a significant effect of gender, we did find that males (relative to females) scored lower on the SMUIS scale. Figure 5 illustrates the marginal effect of BPD trait scores on SMUIS scores.

## Discussion

Impairment in interpersonal functioning has consistently been observed in individuals exhibiting high BPD features^8,9^. The present study explored how this difficulty in interpersonal relationships is expressed in the domain of social media.

First, we explored whether individuals with higher BPD features report greater engagement in social media use. In line with our predictions, higher BPD features were significantly associated with more frequent social media use (P1). Thus, consistent with the finding^14^ that those with a ‘borderline’ profile may be more prone to problematic Facebook use, individuals with higher BPD features in this study may show greater engagement in social media use, plausibly to satisfy their need for social connectedness that may be lacking in the real-world.

Second, we examined whether individuals with higher BPD features are more likely to regret actions on social media. Consistent with our prediction (P2), higher BPD features were significantly associated with more frequent regret posting on social media. This is in line with evidence suggesting that individuals diagnosed with BPD tend to vacillate between seeking and avoiding social connectedness. Over a 20-day period of recording of interpersonal behavior, a BPD clinical group reported significantly greater fluctuations in their levels of agreeableness and quarrelsomeness in their interactions with others, compared to healthy controls^21^. Indeed, it has been suggested^22^ that this tendency to fluctuate between reassurance seeking and interpersonal avoidance may be particularly prominent when individuals with BPD experience perceived rejection from significant others. With this in mind, we may speculate that individuals with high BPD features in the present study may seek social connectedness by posting on social media, but may subsequently regret such actions if the responses (e.g., ‘*likes*’, comments, retweets) from others, or lack thereof, elicit feelings of social rejection. Of course, we cannot draw any firm conclusions about whether these regrets are due to impulsivity or to other features relevant to the structure of BPD, such as a lack of self-worth and clear identity, or a tendency toward experiencing shame, guilt and low self-esteem. Future research should explore this further by examining the reasons for their regret and their ensuing behavior, to determine whether behaviors relating to interpersonal avoidance (e.g., deleting posts) can be observed.

Third, we investigated whether individuals with higher BPD features experience less stable (virtual) social relationships. As predicted (P3), higher BPD features were significantly associated with more frequent unfriending and blocking of other users on social media. This is consistent with the finding^16^ that individuals diagnosed with BPD were more likely to cut-off social partners than non-PD controls. As such, the stormy interpersonal relationships associated with BPD are not limited to real-life interactions but can also be observed in virtual interactions on social media, underscoring the pervasiveness of interpersonal dysfunction associated with BPD. It is important to be cautious, however, as this could also be due to a higher likelihood of people with BPD traits befriending people who are abusive or unhealthy and then getting out of those relationships.

Fourth, we also examined whether individuals with higher BPD features have fewer social connections on social media. Contrary to our prediction (P4), BPD trait scores did not significantly predict the number of friends on Facebook. We did however find that higher BPD features were positively correlated with the number of people following a user on Twitter, and the number of Instagram accounts followed by our participants. Thus, it is noteworthy that those with higher BPD features are able to maintain a network on social media despite more frequent blocking/unfriending. One possible explanation is that social media platforms allow for greater access to forming new social connections than real-life interactions. Alternatively, one study^17^ revealed that individuals diagnosed with BPD were more likely than healthy controls to have conflictual partners who tended to be cut off and then reintroduced into the individual’s social network; social media platforms may facilitate reconciliation (i.e., un-blocking and re-friending) via its various forms of communication (e.g., likes, wave, poke, retweeting) that may seem less daunting than face-to-face reconciliation. We note, however, that we were not able to measure how long our participants owned accounts on each platform. Those who have been active on Facebook for longer, for example, may naturally have more social connections. We might also predict that those with higher BPD features are likely to have been active for longer, given our finding that they post more often. The lack of association observed in this study may reflect variation in time of being active on specific social media platforms.

Finally, we also probed whether people with higher BPD trait scores would spend more time (here, minutes in a day) on social media platforms (P5a), and whether they would report that social media are a more integral part of their lives and daily routines (P5b). Surprisingly, the results did not support the former prediction (P5a) but did support the latter (P5b). It is possible that differences in usage profiles among people with different levels of BPD trait scores manifest rather in a qualitative manner (i.e. how important their life on social media is for them) than in crude quantitative (and self-reported) differences in social media usage time.

There are limitations to the present study, and the results should be interpreted with these in mind. Although this study was the first to demonstrate that interpersonal difficulties associated with BPD features can be observed in social media behavior, future research should explore the motives underlying those behaviors. Exploring the rationalisation for unfriending or blocking other users on social media, for instance, could illuminate the cognitive and affective processes (e.g., inability to repair rupture in relationships) related to such behaviors, which may be useful in informing therapeutic interventions. Additionally, our study relied on self-report retrospective estimations of behavioral frequencies (e.g., regret, blocking/unfriending); future work could employ a prospective diary methodology for a more accurate measurement of social media behavior and the corresponding motives^23^.

A few points of caution are also in order with respect to our sample. First, although there was no mention of BPD in the advertisement for the study, it was stated that participants would be asked to respond to some questions about their experiences with social media. It is therefore possible that our participants were particularly interested in social media and/or that social media is particularly important to them. Secondly, since our participants were recruited from the general population, it is important to note that implications from the present research may not extend to the clinical population, and replication of the studies using clinical samples would be crucial for informing prevention and/or treatment efforts. Thirdly, there are some limitations relevant to online sampling using crowdsourcing (e.g. using Amazon Mechanical Turk). For instance, although online participants tend to produce reliable data when self-reporting on clinically relevant symptoms (e.g., depression, social anxiety), some research^24^ has revealed systematic differences in the demographics and personalities of online participants. For example, one study^25^ indicates that online participants may overestimate psychiatric symptoms. Specifically, the authors found that three percent of their sample scored above the recommended cut-off on the Infrequency-Psychopathology Scale^26^, self-reporting a high frequency of symptoms that tend to be extremely rare in the general population. Nevertheless, one study suggests^25^ that such sites are a useful resource for accessing subclinical (traits) and clinical populations. The prevalence of clinically relevant symptoms such as depression, general anxiety and trauma exposure were comparable to representative samples, while the levels of social anxiety and the prevalence of unemployment and substance abuse problems were higher than in the general population. Moreover, crowdsourcing makes it possible to access a broader sample than the pool of undergraduate students on whom researchers have traditionally depended.

Overall, the present study highlights the pervasiveness of difficulties associated with BPD features in interpersonal functioning even in the non-clinical population. Specifically, we demonstrated that these difficulties in maintaining relationships are not only restricted to face-to-face interactions, but are also observable in social media behavior.

## Method

### Participants

Six hundred and two participants were recruited via Amazon Mechanical Turk. The dataset included 308 female adults (292 males, and two who preferred not to say) aged between 18 to 77 years (*M* age = 39.02 years, *SD* age = 11.51, one participant did not provide their age). We excluded two participants who did not identify as female or male (This exclusion criterion was not pre-registered; it was however decided prior to any analyses, and enabled us to estimate the effect of gender in the subsequent analysis). The experiment was conducted in accordance with the Declaration of Helsinki and was approved by the Humanities & Social Sciences Research Ethics Sub-committee (HSSREC) at the University of Warwick. All participants received a monetary compensation of $0.50.

### Measures

#### BPD features

BPD features were assessed using the short form of the Five-Factor Borderline Inventory (FFBI-SF)^27^. The FFBI-SF is a 48-item self-report measure which assesses BPD features based on the Five Factor Model (FFM) of general personality^28^, and has good internal consistency, convergent, and discriminant validity. FFBI-SF includes four items for each of 12 unique subscales: anxious uncertainty, dysregulated anger, despondence, self-disturbance, behavioral dysregulation, affective dysregulation, fragility, dissociative tendencies, distrust, manipulativeness, oppositional, and rashness. Participants were asked to indicate the extent to which they agree with each statement, using the 5-point scale anchored with “Disagree strongly” and “Agree strongly”. The example items included “*I tend to be quite anxious*”, “*My emotions can spiral out of control*”, and “*I worry a great deal*”. As per previous work and our pre-registration plan, we calculated a single overall score averaging over all items. Higher scores indicate higher the person’s BPD score. To estimate the internal consistency for the total score in the sample, we computed Cronbach’s α = 0.97. The mean FFBI-SF score in our sample was 1.86 (*SD* = 0.76) and ranged from 1 to 4.67. Notably, but expectedly, the scores were also positively skewed with 25% of the sample scoring below the average of 1.21 and 75% scoring below 2.35. In addition to the FFBI-SF, participants were explicitly asked whether they had ever been diagnosed with BPD (21 indicated that they had).

#### Social integration and emotional connection to social media use

The Social Integration and Emotional Connection subscale of Social Media Use Integration Scale (SMUIS) is a 6-item subscale (anchored with Strongly disagree and Strongly agree) that measures the extent to which usage of social media is integrated into social behavior, as well as the importance of and one’s emotional connection to this use^29^. Example items are ‘*Facebook plays an important role in my social relationships*’ and ‘*I feel disconnected from friends when I have not logged into Facebook*’. Internal consistency for this subscale in this sample was high, with Cronbach’s α = 0.91. Overall, the mean score of our sample was 3.13 (*SD* = 0.86) and ranged from 1 to 5.

#### Social media behavior

In order to assess social media use, participants were asked a series of questions about their social circle on popular social media platforms. First, participants were asked about the average amount of time (in minutes) that they spend in a typical day on each of the following social media platforms: Facebook, Twitter, Snapchat, Instagram, Tumblr, Reddit and Pinterest. Unless participants typed 0, they were also asked to report the number of friends they have on Facebook, and the number of followers (people who follow the participants’ profile) as well as followings (profiles the participants follow) on Instagram and Twitter.

Additionally, to assess the behavioral frequency of social media use, participants were asked to report how often in the past two weeks have they (a) posted on social media, (b) regretted posting on social media, (c) edited or deleted something they posted on social media, (d) unfriended, blocked or muted someone, and (e) re-friended, un-blocked or un-muted someone. Finally, participants also reported how many new friends or individuals did they begin to follow in the past two weeks. For all of the above questions participants could respond by typing any number in the box.

### Procedure

Before completing the measures listed above, participants provided informed consent and indicated their age and gender. Next, participants answered questions about their social media use, social and emotional integration of social media, and BPD traits, in that order. At the end of the study, participants were asked whether they received a diagnosis of BPD in the past. All participants were thanked for their participation and asked to provide their Mturk ID to evidence their completion of the survey.
